# Milk-Clotting Properties and Primary Proteolysis of a Lyophilised Extract from Artichoke Flowers (*Cynara scolymus* L.)

**DOI:** 10.17113/ftb.61.03.23.8142

**Published:** 2023-09

**Authors:** Valentina Crosetti, Agustín Sola, Gabriela Grigioni, María José Torres

**Affiliations:** 1 Department of Basic and Experimental Sciences, National University of the Northwest of the Province of Buenos Aires (UNNOBA), Newbery 355, 6000 Junín, Buenos Aires, Argentina; 2 Research and Transfer Center of the Northwest of the Province of Buenos Aires (CITNOBA) – UNNOBA-UNSadA-CONICET, Monteagudo 2772, 2700 Pergamino, Buenos Aires, Argentina; 3 Institute of Food Technology - Institute of Science and Technology of Sustainable Food Systems, INTA CONICET, CC 25, 1712 Castelar, Buenos Aires, Argentina

**Keywords:** *Cynara scolymus*, lyophilised vegetable coagulant, clotting activity, casein proteolysis, plant rennet

## Abstract

**Research background:**

A few studies have investigated *Cynara scolymus* enzymes as a substitute for calf rennet in cheese making. They used aqueous extracts prepared by maceration of plant material and stored by freezing. However, it was indicated that lyophilisation is a better alternative to preserve the coagulant properties of plant extracts over a longer period of time, as it is a more controllable, stable and hygienic alternative with a better shelf life that is easier to transport, store and standardise.

**Experimental approach:**

We obtained a lyophilised extract of mature artichoke flowers, named CS, which was characterised for its milk-clotting properties at different pH and temperatures. In addition, the potential yield, whey composition and the primary hydrolysis profile of caseins by urea-polyacrylamide gel electrophoresis (PAGE) of mini curds prepared with different doses of coagulant were determined.

**Results and conclusions:**

The lyophilised extract was able to hydrolyse casein and showed stable proteolytic activity at pH=6.4 and 37 °C for 50 min, which decreased when the process temperature was increased to 41 and 45 °C and was lost at 70 °C. On the other hand, milk-clotting activity increased significantly (p<0.001) when the temperature increased from 37 to 45 °C and the pH of the milk decreased from 6.8 to 5.8. Potential yield ​​between 10 and 17 % was obtained for the mini curds prepared with the lyophilised artichoke extract, and the casein degradation pattern obtained by urea-PAGE was similar to that of the commercial coagulant.

**Novelty and scientific contribution:**

On a laboratory scale, our work has shown that the lyophilised artichoke extract has sufficient proteolytic and coagulant activity to be used as a milk coagulant, *i.e*. plant rennet, in cheese making as an alternative to animal rennet. As this extract is lyophilised, it has the advantage of being a better alternative in terms of preservation and shelf-life. It offers an innovative way to diversify cheese products and appeal to consumers with different dietary preferences and needs.

## INTRODUCTION

The increasing global demand for cheese and consumer interest in new flavour and texture alternatives encourage the search for new milk coagulants to replace animal rennet in cheese production. In this context, alternatives from various sources, including microbial and plant sources, have been investigated. Microbial coagulants have proven to be a viable alternative thanks to their high milk-clotting activity and low production costs. However, some of them have certain disadvantages, such as excessive proteolysis and high thermostability, which affect the sensory properties of the cheese and produce a bitter taste (*1*). On the other hand, crude aqueous extracts of several vegetable species have also been studied as possible substitutes (*2*-*4*). These extracts are usually prepared by macerating plant material and then stored refrigerated or frozen until use. However, several studies have pointed to lyophilisation (or freeze-drying) as a better alternative to preserve the coagulant properties over a longer period of time (*5*-*7*). Freeze-drying has been studied for the preservation of microorganisms, nanoparticle systems, nucleic acids and proteins, as the aqueous solutions of these components have low stability and short half-lives (*8*). Therefore, they must be stored frozen, which takes up a larger volume and causes greater inconvenience or logistical costs.

Studies conducted with cardoon (*Cynara cardunculus* L.) extract have shown that the coagulation of the extract decreased after 4 to 5 months of frozen storage, while the coagulation of the lyophilised extract remained constant for at least 1 year and viable microorganisms disappeared after freeze-drying (*5*). Furthermore, in a study with the coagulant extract from *Whitania* an overall decrease in activity of 23 % was observed in the extract stored frozen, while the lyophilised extract maintained the highest milk coagulation activity and lost only 6 % of activity after 150 days of storage (*6*). Another study showed that the clotting ability of the liquid extract of *Ficus carica* decreased significantly after 12 months of cold storage at 4 °C, while the lyophilised extract retained its full clotting potential (*7*). In general, authors agree that refrigerated storage is not suitable for long preservation of aqueous extracts as it prolongs the coagulation time and increases microbial contamination as well as the deterioration of organoleptic properties. Conversely, lyophilisation provides a more stable, controllable and hygienic alternative for the preservation of the vegetable coagulant with better shelf life and easier transport, storage and standardisation.

The flowers of the genus *Cynara* contain aspartic proteases with high milk-coagulating power, which can be used to produce cheese with a creamier and smoother texture and more intense aroma and flavour than when using commercial chymosin or calf rennet (*9*). Extracts from *Cynara scolymus* are able to hydrolyse κ-casein and have shown milk coagulation properties (*4*, *9*-*12*). However, the few studies conducted so far have used aqueous extracts, which have the drawbacks mentioned before. Based on the above, the objective of this study is to obtain and characterise a lyophilised plant coagulant from mature artichoke flowers as a substitute for commercial rennet. Here, we characterised the milk-coagulating properties, protein content and proteolytic activity of the extract on casein. We also determined the potential yield and the α_s_- and β-casein degradation profile of miniature curds prepared with this vegetable coagulant. All tests were performed in comparison with commercial coagulants.

## MATERIALS AND METHODS

### Plant material and preparation of the lyophilised artichoke extract

The samples used in this study were pistils of artichoke (*Cynara scolymus*) flowers obtained from the experimental field of the National University of the Northwest of the Province of Buenos Aires, Buenos Aires, Argentina, and collected in January 2021, when the plant is ripe and dry. Ten flowers were collected and stored at 25 °C in a dark and dry environment for one day for later processing.

The extracts were prepared according to the method of Llorente *et al*. (*12*), with some modifications. An aliquot of 5 g pistils was crushed by hand in a mortar with the addition of liquid nitrogen and macerated in an ice bath containing 250 mL of 0.1 M phosphate buffer, pH=7 (Biopack, Buenos Aires, Argentina) and 5 mM EDTA (Cicarelli, Santa Fe, Argentina) for 40 min. The mixture was filtered through double gauze and then centrifuged at 3500×*g* (Neofuge 23R; Heal Force, Shanghai, PR China) for 20 min. The supernatant was fractionated and lyophilised for 7 h at -55 °C and 0.09 mPa in a freeze dryer (MPS-55; Operon, Gyeonggi, Korea). The lyophilised powder obtained was designated CS.

### Characterisation of the lyophilised artichoke extract

#### Determination of proteolytic activity

The proteolytic activity was determined according to the method described by Torres *et al*. (*13*), with some modifications. The reaction mixture was prepared with 1.1 mL bovine casein (Sigma-Aldrich, Merck, St. Louis, MO, USA), 1 % (*m*/*V*) of 0.1 M phosphate buffer (pH=5.8–6.8) containing 5 mM EDTA and 0.1 mL lyophilised powder resuspended in distilled water. The mixture was incubated at 37, 45, 55 and 60 °C, and stopped after 30 min with 5 % (*m*/*V*) trichloroacetic acid (Biopack, Buenos Aires, Argentina). Samples were centrifuged at 2000×*g* for 5 min and the absorbance of the supernatant was measured at 280 nm (UV/Vis spectrophotometer SP-2000UV; Mettler Toledo, Columbus, OH, USA) to determine the content of soluble peptides obtained by hydrolysis of bovine casein. Proteolytic activity was expressed as caseinolytic units (Ucas), *i.e*. an arbitrary unit corresponding to the amount of enzyme required to increase the absorbance unit by 1 at 280 nm after 1 min of reaction (*14*).

#### Determination of the protein concentration

The Bradford (*15*) method was used to determine the protein concentration of the lyophilised powder resuspended in distilled water. The assay was performed in triplicate, placing 10 µL of the sample on a plate together with 300 µL Bradford reagent containing Coomassie Brilliant Blue G-250 (Sigma-Aldrich, Merck). After allowing the complex to stabilise for 20 min, the absorbance at 595 nm was measured using a microplate absorbance reader (iMarkTM; Bio-Rad, Hercules, CA, USA). The calibration curve was made in the range of 0.05 to 1 mg/mL using bovine serum albumin under the same conditions as for the sample.

#### Thermal stability and inactivation

To evaluate thermal stability, the residual activity (%) was determined as the remaining proteolytic activity after a previous heat treatment of the lyophilised powder at different temperatures (37, 45, 55 and 65 °C) for 15, 30, 45, 60, 90, 120, 150 and 180 min. For thermal inactivation, heat treatments at 50, 60 and 70 °C for 2, 5 and 7 min were used.

#### Influence of pH and temperature on milk clotting activity

The clotting time was evaluated using skimmed bovine milk powder (protein 2.9 and fat 0.1 % (*m*/*V*), Purísima, La Sibila SA, Entre Ríos, Argentina) reconstituted to 10 % (*m*/*V*) with 10 mM CaCl_2_ by adjusting the pH with lactic acid and setting the temperature of the water bath. The time to the initial appearance of solid material was recorded and the clotting units were calculated as the inverse of the average of these times (*13*). All the assays were performed in triplicate and compared with a commercial coagulant (FAR-M© sticks, Chr. Hansen, Hørsholm, Denmark) under the same experimental conditions. The commercial coagulant is a recombinant camel chymosin, which has higher clotting activity for bovine milk and lower proteolytic activity than bovine chymosin, as reported by Kappeler *et al*. (*16*).

### Potential curd yield and whey composition

We prepared miniature curds in 50-mL centrifuge tubes containing 40 mL powdered skimmed bovine milk (Purísima) reconstituted to 10 % (*m*/*V*) with 0.2 g/L CaCl_2_ and acidified with lactic acid at pH=6.4 (optimal pH determined for the lyophilised extract in this study) to simulate the effect of the fermenter that is usually added before the coagulant, which causes a decrease in the pH due to the formation of lactic acid by the metabolisation of lactose by lactic acid bacteria. Two coagulants were used: lyophilised powder (CS) resuspended in distilled water at two concentrations: 3.4 and 1.3 mg protein per mL extract and commercial coagulant (CC) prepared with similar clotting units as the lyophilised powder: 0.14±0.03. These coagulants were added in two volume fractions (50 and 75 µL extract per 10 mL milk). The samples were thus designated: CS 3.4-50, CS 3.4-75, CS 1.3-50, CS 1.3-75, CC-50 and CC-75; each sample was prepared in triplicate. The contents of the tubes were mixed well and quickly placed in a water bath at the same temperatures used for milk clotting activity (37, 41 and 45 °C). Coagulation time was determined subjectively as the time when casein flocculation was visually observed. The tubes were placed back in the water bath for a period of time equal to that of coagulation time and the clot was cut into small cubes with a stainless steel spatula. They were then kept in the bath for a further 15 min to encourage draining and then centrifuged at 10 000×*g* (Neofuge 23R; Heal Force) for 15 min to simulate and standardise the pressure exerted during moulding (*17*).

The potential curd yield (%) of each sample was calculated as the ratio between the coagulum mass and the milk mass (kg of curd per 100 kg of milk). Whey composition was determined using an ultrasonic analyser (Lactoscan, Milkotronic, Nova Zagora, Bulgaria).

### Analysis of casein hydrolysis by urea-PAGE

The proteolytic activity of the prepared mini curds was evaluated using urea-PAGE with casein as a control. Samples were prepared according to the method described in detail by Llorente (*18*), with the following modification: sample buffer was adjusted to the protein content of each sample previously determined by the Bradford method (*15*). Then, 4 µL of sample were seeded and the measurement was carried out in a Mini-Protean IV Cell (Bio-Rad, Hercules, CA, USA) for 40 min at constant voltage using a power source (Labnet, Windsor, UK). The gels were stained with colloidal Coomassie G-250 (Sigma-Aldrich, Merck) (*13*, *19*). The α- and β-caseins were quantified by densitometry, by scanning the gels and quantifying the intensity of the protein bands using ImageJ software (*20*).

### Statistical analysis

Statistical analysis was conducted using InfoStat software 2020 e-version (*21*). Data normality was checked with the Shapiro-Wilk’s test and homogeneity of variances with Levene’s test. Data that did not show a normal distribution or homogeneity of variances (p<0.05) were analysed with the Kruskal-Wallis’s test. ANOVA was used to compare the effects of temperature and pH on the clotting time and proteolytic activity, temperature and time on thermal stability, the amount of coagulant on potential curd yield and degradation of the α- and β-casein fractions. Tukey’s test was used to evaluate differences between samples at a 95 % confidence level.

## RESULTS AND DISCUSSION

**Fig. 1:**
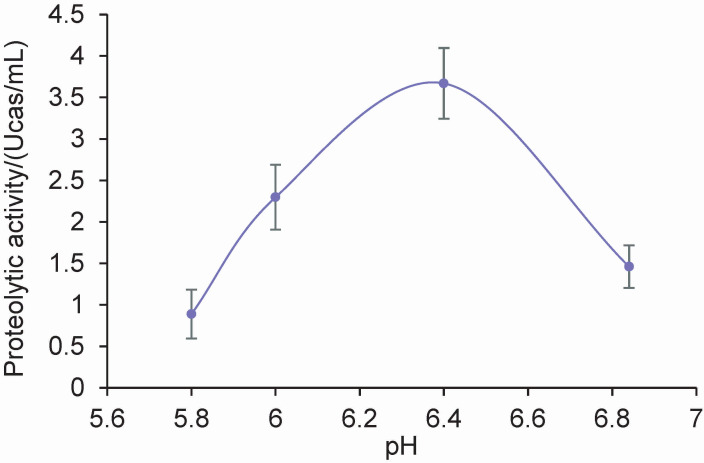
The effect of pH on the hydrolysis of casein by artichoke coagulant expressed in Ucas (caseinolytic units) per mL of enzyme extract. Data points represent the mean value of triplicate tests, and each experiment was repeated twice. Error bars represent mean±standard deviation

An enzymatic extract (here designated CS) with a proteolytic activity on casein at 37 °C and pH values between 5.8 and 6.8 was obtained from the pistils of the artichoke flower, with higher values measured at pH=6.4 (p<0.001) (Fig. 1). The protein concentration, determined by the Bradford method (*15*), was (0.89±0.04) mg/mL, and the specific activity of enzymatic extract on casein at pH=6.4 (obtained by dividing the proteolytic activity by the protein concentration) was (0.19±0.01) Ucas/mg. It should be noted that the proteolytic activity of lyophilised powder is lower than that of crude extracts from other plant sources such as the crude extracts of the ripe fruits of *Vasconcella quercifolia* (3.4 Ucas/mg) (*13*), *Bromelia hieronymi* ((10.9±2.2) Ucas/mg) (*22*) and *Maclura pomífera* ((4.1±0.2) Ucas/mg) (*23*). Also, the specific activity of lyophilised powder was lower than that of a crude aqueous extract of the mature flower of artichoke, which was (5.45±0.09) Ucas/mg (*24*). The moderate proteolytic activity of lyophilised powder can be considered a positive characteristic, especially in cheese production since excessive proteolysis leads to loss of firmness of the curd and the development of undesirable bitter flavours (*25*).

**Fig. 2:**
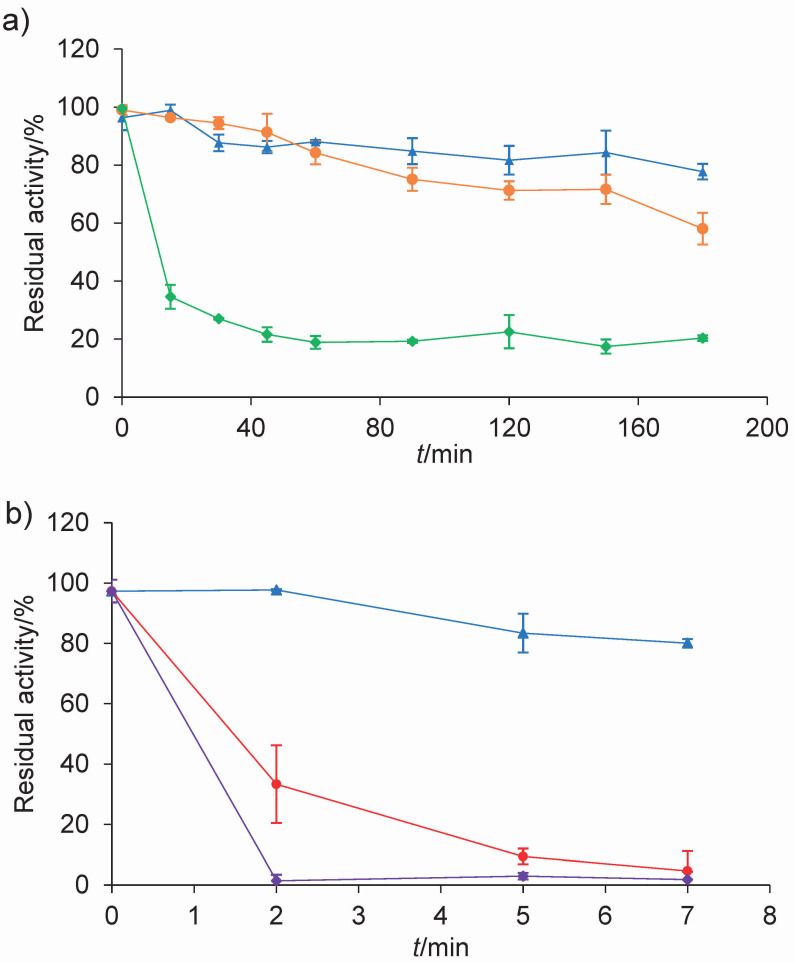
The evaluation of: a) thermal stability of the artichoke coagulant at 37 (blue), 45 (orange) and 55 °C (green). Data are means of three determinations and b) inactivation of the artichoke coagulant at 50 (blue), 60 (red) and 70 °C (purple). Data are means of duplicate tests. Error bars represent mean±standard deviation. Residual activity (%) was determined on casein at pH=6.4

Our investigation also included a thermal stability and inactivation assay, the results of which are shown in Fig. 2. When analysing the residual activity of the extracts, a significant interaction between time and temperature was found (p<0.001). At 37 and 45 °C, the residual activity remained above 80 % for up to 45 min, without differences between the two treatments (Fig. 2a). This represents an advantage when using extracts as coagulants because the coagulation time in cheese making is approx. 40 to 50 min. Thus, lyophilised powder was able to maintain its activity during the processing time. Differences began to appear after 60 min, (p<0.001), while after 3 h the activity remained practically unchanged at 37 °C, whereas at 45 °C it was reduced by 51 %. On the other hand, the extract seems to be a thermolabile enzyme since 60 % of the activity at 55 °C was lost after 15 min and no residual activity was observed at 65 °C at the times tested (data not shown). These results are in agreement with those of Llorente *et al.* (*25*), who tested an aqueous extract of artichoke at pH=6. Its proteolytic activity remained practically unchanged for 3 h at 37 °C and exceeded 70 % at 45 °C. These authors also found that at 55 °C the extract lost 60 % of its activity after only 40 min and that at 65 °C it was still not completely inactivated. These results suggest the stability of the extract under different production conditions and support its potential as a coagulant in cheese production.

In the thermal inactivation test (Fig. 2b), total loss of proteolytic activity was observed when heated at 70 °C for 2, 5 and 7 min and at 60 °C for 7 min, while no significant decrease in the activity was evidenced when heated at 50 °C for 7 min. The proteolytic activity of the coagulating enzyme is an important property to consider when looking for substitutes for calf rennet. One of the crucial characteristics to assess their applicability is that they have high milk coagulating activity (MCA) and low proteolytic activity (PA) (*26*). A high MCA/PA ratio allows the production of cheeses with adequate sensory and textural properties, high yields and a reduced production of peptides that give the cheese a bitter taste (*24*). The low specific proteolytic activity observed in our extract (lyophilised powder) along with its heat sensitivity could offer interesting advantages in the control of flavour defects that may occur during cheese production: for example, the inclusion of heating steps in the production process to reduce residual proteolytic activity. It also offers an additional advantage over other calf rennet substitutes on the market, such as microbial coagulants. Although these coagulants have been proven to be effective for cheese making, they used to have high thermal stability and an excessive proteolysis on bovine casein, such as the coagulants obtained from *Rhizomucor miehei*, *Mucor pusillus* and *Bacillus polymyxa*, among others. Strong thermal stability increases the negative effects of residual activity on cheese texture and flavour during cheese ripening (*1*).

**Fig. 3:**
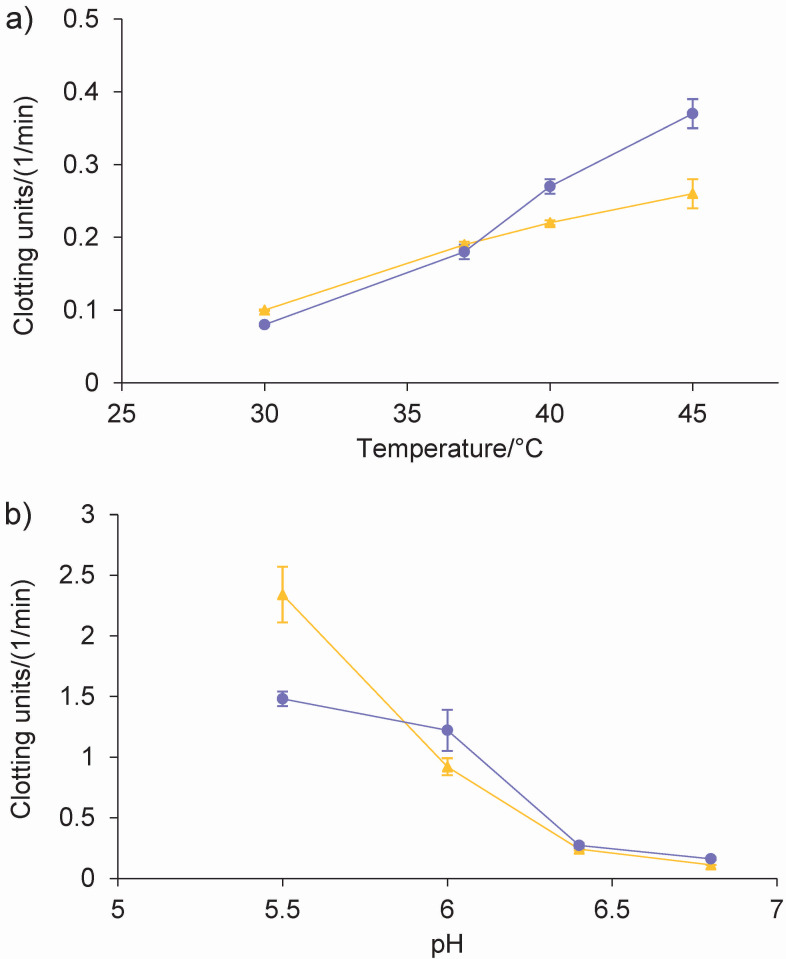
Comparison of changes in coagulation time (measured as number of clotting units (CU)) using artichoke coagulant (purple) and commercial rennet (yellow) as a function of: a) temperature and b) pH. The number of CU was calculated as the inverse of the average of clotting time expressed in minutes. Error bars represent mean±standard deviation. Data are means of triplicate tests

The coagulation activity assays showed significant interaction between the main factors (p<0.001). The results showed that when the temperature increased from 37 to 45 °C and milk pH decreased from 6.8 to 5.8, the activity measured in clotting units (CU/(1/min)) significantly increased (p<0.001) for both coagulants (Fig. 3a and Fig. 3b). Under the conditions tested, the milk-clotting activity of both coagulants remained similar at 30 and 37 °C, whereas lyophilised powder had higher values than the commercial coagulant at 40 and 45 °C (Fig. 3a). At pH=5.5, the CU (1/min) of the commercial coagulant was significantly higher than that of the lyophilised powder, while no significant differences were found at the other pH values tested (Fig. 3b). In a study with an aqueous artichoke extract, Chazarra *et al.* (*11*) reported a progressive reduction of clotting time from 20 to 60 °C and found that the coagulation process slows down at higher temperatures. These authors also observed that an increase in the pH of the milk was accompanied by a loss of the milk-clotting activity of the extract, and that the extract lost 87 % of its activity at pH=7. Although a high CU (1/min) was obtained at 40 and 45 °C for lyophilised powder, the cheese coagulation temperature ranges from 32 to 38 °C depending on the type of maturation (*27*). These findings suggest that lyophilised powder could serve as an effective coagulant for milk under conditions resembling the production of soft cheese with relatively short ripening periods (20 to 30 days). This potential use aligns with cheese-making practices for varieties such as Quartirolo: coagulation at 38 °C and pH=6.4 (*28*), Cremoso and Port Salut cheeses: coagulation at 37-40 °C and pH=6.4 (*29*). Future research is needed to explore the practical applications and sensory characteristics of the cheese produced using lyophilised powder as a coagulant.

**Fig. 4:**
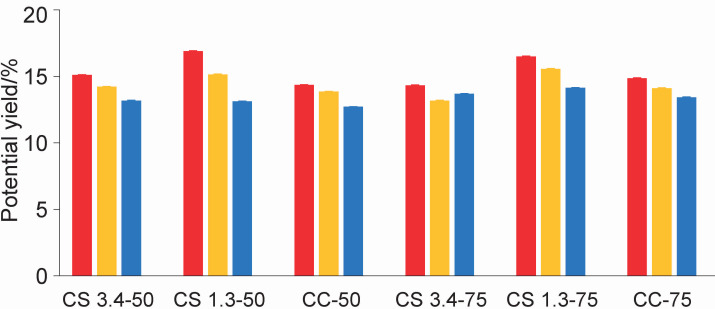
Potential yield of mini curds made using different doses and concentrations of the artichoke extract lyophilised powder (CS) and commercial coagulant (CC) at 37 (red bars), 41 (yellow bars) and 45 °C (blue bars). Error bars represent mean±standard deviation. Data are means of triplicate tests. 3.4 and 1.3=protein concentration of the extract (mg/mL), 50 and 75=volume of extract per 10 mL of milk

The assay with miniature curds showed that the coagulation times decreased as temperature increased: 65 min at 37 °C, 50 min at 41 °C and 40 min at 45 °C. No significant interaction was observed in potential curd yield between the main factors. Fig. 4 shows that the yield decreased significantly when the coagulation temperature increased from 37 to 45 °C (p<0.001). These results are consistent with those of Madrid (*30*), who explain that although fast coagulation is obtained at high process temperatures, this leads to the formation of inadequate curd with the consequent loss of performance and quality. Sample CS 1.3 produced a higher yield than the commercial coagulant at both evaluated amounts. While sample CS 1.3-75 had a significantly higher yield (p<0.001) than all other samples, it should be assessed in the scaling up of the process whether using a dose higher than 50 % is justified, as CS 1.3-50 gave an acceptable yield higher than that of CC-50.

The results in Table 1 indicate that as the coagulation temperature increased, the mass fractions of protein, lactose and non-fat solids in the whey and its density obtained from mini curds after centrifugation decreased. Similarly, when comparing the commercial coagulant with lyophilised powder, we observed higher mass fractions of these components in the lyophilised powder. Additionally, a decrease in the mass fractions of protein, lactose and non-fat solids is observed with lower amounts of lyophilised powder. However, in no case the differences are statistically significant. Furthermore, when these results are analysed in the context of the findings shown in Fig. 4, the decrease in the yield could be associated with a higher loss of water from the mini curd, which is evidenced by observing the values ​​of water in whey (Table 1). In addition, the higher amount of water in the whey could explain the reduction in the mass fraction of the other measured components. Based on the findings at the laboratory scale (technology readiness level (TRL) 2) so far (*31*), the conditions of 37 °C and pH=6.4 would be suitable to initiate an optimisation process on a larger scale. Although the coagulation time at 37 °C was longer, the mini curds had greater firmness and yield, which is highly valued by the cheese industry.

**Table 1:** The composition and density of the whey obtained from mini curds at 37, 41 and 45 °C determined with an ultrasonic milk analyser Lactoscan

Whey composition and density	Temperature/°C						
CS 3.4-50	CS 1.3-50	CC-50	CS 3.4-75	CS 1.3-75	CC-75
*w*(protein)/%	37	2.88	2.81	2.73	2.86	2.80	2.72
	41	2.78	2.76	2.72	2.82	2.77	2.70
	45	2.78	2.73	2.70	2.80	2.75	2.68
*w*(lactose)/%	37 41 45	4.29	4.18	4.07	4.28	4.15	4.06
		4.14	4.09	4.05	4.20	4.13	4.01
		4.14	4.05	4.03	4.18	4.10	4.02
*w*(non-fat solid)/%	37	7.80	7.62	7.40	7.80	7.57	7.39
	41	7.54	7.46	7.37	7.66	7.52	7.31
	45	7.55	7.38	7.33	7.61	7.45	7.33
*ρ*/%	37	31.50	30.48	29.71	31.29	30.29	29.62
	41	30.10	29.84	29.45	30.56	29.96	29.33
	45	30.12	29.39	29.22	30.31	29.63	29.19
*w*(water)/%	37	8.97	11.34	14.04	9.03	11.73	14.23
	41	11.83	13.33	14.42	10.58	12.50	15.28
	45	12.18	14.35	14.90	11.47	13.39	14.90

Data are means of triplicate tests. All S.D. are less than 0.01. CS=artichoke coagulant, CC=commercial rennet, 3.4 and 1.3=protein concentration of the extract (mg/mL), 50 and 75=volume of extract per 10 mL of milk

**Fig. 5:**
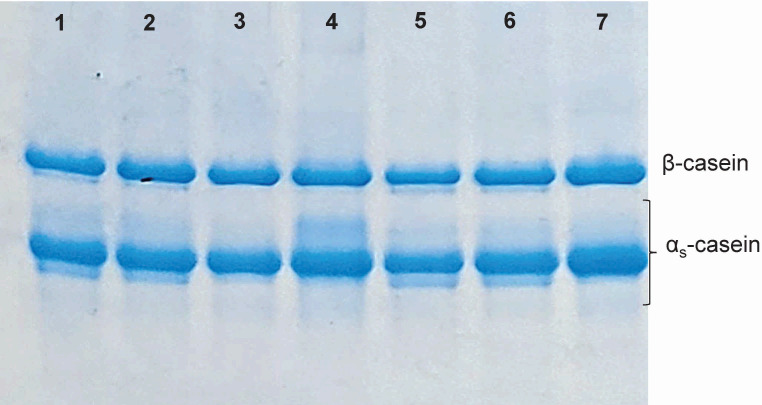
Urea-polyacrylamide gel electrophoresis (PAGE) of miniature curds made at 37 °C. Lane 1=CS 3.4-50, lane 2=CS 1.3-50, lane 3=CC-50, lane 4=standard casein, lane 5=CS 3.4-75, lane 6=CS 1.3-75 and lane 7=CC75. CS=artichoke coagulant, CC=commercial rennet, 3.4 and 1.3=protein concentration of the extract (mg/mL), 50 and 75=volume of extract per 10 mL of milk

Finally, to complement the characterisation of the extract, the hydrolysis of casein subunits was analysed by urea-PAGE in the mini curds obtained under different conditions (coagulant amounts/temperature) in duplicate. Fig. 5 shows the primary proteolysis of mini curds at 37 °C (images of that observed at 41 and 45 °C are not shown). Initially, the bands of α_s_- and β-casein subunits were visually identified with urea-PAGE electrophoretograms obtained under comparable experimental conditions published in the paper by Milesi *et al.* (*32*). In our gel, the appearance of bands with higher mobility can be observed in the samples of the mini curds obtained with lyophilised powder than those obtained with commercial coagulant. These results suggest that the lyophilised powder may induce a higher degree of casein hydrolysis than the commercial coagulant, potentially influencing the sensory characteristics of the final product. However, Table 2 shows the densitometry analysis of the gels, where the relative intensity of the bands corresponding to the α_s_- and β-casein fractions was calculated for each treatment, comparing the degree of degradation to the casein control. No significant interaction was observed between coagulation temperature and type of coagulant. The degradation of the β-casein fraction was lower than 3.5 % in all the treatments tested, without statistically significant differences. The analysis of the α_s_-casein fraction showed a significantly higher degradation at 41 °C, without significant differences when changing the type and amount of coagulant. With these results we can show that although more mobility bands corresponding to hydrolysis products are observed when using the lyophilised powder, the intensity does not reveal a significantly higher hydrolysis effect of lyophilised powder than of commercial coagulant. This is important because previous research has shown that one of the most frequent off-flavour problems in cheese is bitterness, associated with the production of peptides with hydrophobic amino acid residues originating from excessive casein hydrolysis, mainly α_s_1- and β-casein (*32*, *33*). Based on this, to continue characterising the coagulant here obtained from *C. scolymus* (artichoke) flowers for its use as a substitute in cheese making, it would be advisable to evaluate the residual activity of the enzyme during ripening to see the impact on the degradation of casein subunits.

**Table 2:** Degradation of α_s_- and β-casein subunits: residual fraction (%) referred to the initial content of casein control (100 %)

Casein	Temperature/°C							
CS 3.4-50	CS 1.3-50	CS-50	CS 3.4-75	CS 1.3-75		CC-75
		Residual subunit/%				
α_s_	37	97.3^aA^	98.1^aA^	98.2^aA^	100^aA^	99.0^aA^	100^aA^	
	41	95.5^aB^	96.5^aB^	97.1^aB^	93.7^aB^	96.0^aB^	97.6^aB^	
	45	96.7^aA^	99.7^aA^	99.8^aA^	97.8^aA^	98.2^aA^	99.3^aA^	
β	37	96.5^aA^	97.5^aA^	98.7^aA^	97.5^aA^	99.3^aA^	98.5^aA^	
	41	96.9^aA^	97.4^aA^	99.2^aA^	97.9^aA^	98.7^aA^	98.4^aA^	
	45	97.0^aA^	97.1^aA^	99.0^aA^	99.3^aA^	99.8^aA^	99.4^aA^	

Data are means of triplicate tests. All S.D. are less than 0.01. Means with the same letter in superscript are not significantly different (p>0.05). Capital letters refer to temperature and lower-case letters refer to the type of coagulant. CS=artichoke coagulant, CC=commercial rennet, 3.4 and 1.3=protein concentration of the extract (mg/mL), 50 and 75=volume of extract per 10 mL of milk

## CONCLUSIONS

At laboratory scale, our work demonstrated that the lyophilised artichoke extract (CS) has adequate proteolytic and coagulant activity to be used as a milk coagulant in cheese making. Also, the mini curd assays suggest that this extract can be used as a coagulant in soft cheese making, given that the highest yield was obtained at 37 °C. Additionally, the lowest concentration of extract (protein 1.3 mg/mL) obtained the highest yield when added in volumes of 50 and 75 µL per 10 mL of milk (samples: CS 1.3-50 and CS 1.3-75), without a significant impact in the degradation of the α_s_- and β-casein fractions. However, when scaling up the process, it will be necessary to evaluate the sensory, chemical and functional properties of the produced cheese and its acceptability by the consumers. This research paves the way for innovations in cheese production, offering an alternative to diversify the product and appeal to consumer with different dietary preferences and concerns. Such innovations can contribute to the sustainable evolution of the cheese industry while meeting the various demands of today's consumers.
